# Activity of *Culicoides* spp. (Diptera: Ceratopogonidae) inside and outside of livestock stables in late winter and spring

**DOI:** 10.1007/s00436-016-5361-2

**Published:** 2017-01-04

**Authors:** Daniela Kameke, Helge Kampen, Doreen Walther

**Affiliations:** 1 Institute of Land Use Systems, Leibniz Centre for Agricultural Landscape Research (ZALF), Eberswalder Str. 84, 15374 Müncheberg, Germany; 2 Institute of Infectology, Friedrich-Loeffler-Institute (FLI), Südufer 10, 17493 Greifswald, Germany

**Keywords:** Overwintering, Biting midges, Farms, Winter phenology, Vectors, Arboviruses

## Abstract

*Culicoides* Latreille, 1809 midge species are the putative vectors of Bluetongue virus (BTV) and Schmallenberg virus (SBV) in Europe. To gain a better understanding of the epidemiology of the diseases, basic knowledge about the overwintering of the vectors is needed. Therefore, we investigated culicoid activity in relation to air temperature at livestock stables during late winter and spring season. Ceratopogonids were captured weekly indoors and outdoors on three cattle farms, three horse farms and one sheep farm in the federal state of Brandenburg, Germany between January and May, 2015 by BG-Sentinel UV-light suction traps. First seasonal activity was measured inside a sheep barn and cattle stables in mid-March, suggesting the existence of a preceding vector-free period. The first species at all trapping sites were members of the Obsoletus Complex followed by *Culicoides punctatus* (Meigen), 1804 and *Culicoides pulicaris* (Linnaeus), 1758 simultaneously. In total, 160 collections were made, including 3465 *Culicoides* specimens with 2790 (80.6%) of them being members of the Obsoletus Complex. The remaining 675 individuals belonged to six other culicoid species. 59.8% of all *Culicoides* were collected indoors, and almost five times as many midges were sampled on cattle farms as on horse farms. Cattle farms harboured seven species while only two species were found on the horse and the sheep farms, respectively. Temperatures, husbandry practises and the presence/quality of potential breeding sites might be responsible for the difference in species and numbers of caught specimens between livestock holdings.

## Introduction

Since the outbreak of bluetongue disease in Central and northern Europe in 2006, the interest attracted by culicoid biting midges (Diptera: Ceratopogonidae) as virus vectors has increased significantly. These dipteran insects are known to transmit numerous pathogens, including Bluetongue virus (BTV) (genus *Orbivirus*, family *Reoviridae*) and Schmallenberg virus (genus *Orthobunyavirus*, family *Bunyaviridae*). Both viruses are known to be transmitted by several culicoid species such as members of the Obsoletus Complex (De Regge et al. 2012; Hoffmann et al. 2009; Mehlhorn et al. 2007; Rasmussen et al. 2012) and some species of the Pulicaris Group (Hoffmann et al. 2009; Larska et al. 2013).

After their first appearance in Central and northern Europe, both bluetongue and Schmallenberg disease re-emerged in consecutive years in several European countries (Conraths et al. 2013; Hoffmann et al. 2008), infecting ruminants and causing high losses to the farming industry. The recurrence of both diseases gave reason to suggest that their pathogens were able to survive the cold winter months (Doceul et al. 2013; EFSA 2012; Hoffmann et al. 2009). Up to this day, the exact mechanisms underlying the overwintering of BTV and Schmallenberg virus (SBV) in temperate zones have remained unclear, though (EFSA 2014; Napp et al. 2011).

The persistence of the viruses within their adult biological vectors is considered by many to be the most likely pathway of overwintering (Doceul et al. 2013; Goffredo et al. 2013; Hoffmann et al. 2008; Larska et al. 2013; Losson et al. 2007; Mehlhorn et al. 2009a, b; Tarlinton et al. 2012).

Initially, most biting midge species of the genus *Culicoides* Latreille, 1809 had been believed to outlast the winter months as larvae (Mellor et al. 2000; Rawlings and Mellor 1994; Rowley 1967). Due to the recurrence of BTV, followed by acute SBV infections during winter (Wernike et al. 2013), it was soon speculated that a constant adult midge population might remain active throughout the coldest months of the year (Hoffmann et al. 2008; Losson et al. 2007; Tarlinton et al. 2012). Some studies soon confirmed permanent midge activity during winter even in temperate regions (Hoffmann et al. 2009; Mehlhorn et al. 2009a, b). Critical voices, however, do not believe that the low adult midge population during winter is able to keep the virus circulation alive until the start of the next vector season. Therefore they suggest, that other, so far undetected pathways, might exist which can explain the persistence of viruses (Napp et al. 2011).

The present study was conducted to provide baseline information on the winter phenology of biting midges near livestock animals. We aimed to determine the culicoid species composition inside and outside of different livestock stables during late winter and spring and try to identify (species-specific) temperatures which initiate midge activity.

## Materials and methods

### Study period, area and sites

Insect collections took place between 25 January and 10 May 2015 at three cattle stables and three horse stables. Additionally, one stable harbouring sheep was tested between mid-March and May, 2015. All stables were located in the German federal state of Brandenburg in two regions ca. 50 km apart from each other (Table 1) and sampled on an almost weekly basis.

**Table 1:** Location and number of collections at all sampling sites

Farm	Coordinates	Site	No. of samples
Region 1			
Horse 1	N 52.363679	Inside	10
	E 13.391921	Outside	10
Horse 2	N 52.375751	Inside	13
	E 13.367597	Outside	13
Horse 3	N 52.398795	Inside	10
	E 13.447769	Outside	10
Region 2			
Cattle 1	N 52.541143	Inside	14
	E 14.18044	Outside	14
Cattle 2	N 52.541157	Inside	14
	E 14.168886	Outside	14
Cattle 3	N 52.567115	Inside	11
	E 14.231117	Outside	11
Sheep	N 52.513221	Inside	8
	E 14.17472	Outside	8

All stables were of solid structure and shut for the most part, but contained permanent openings useable as entrances for flying insects. They harboured exclusively cattle, horses or sheep with >5 host animals being continuously kept inside the stables. More animals of the same species were present outside at all times, belonging either to the barnyard of examination or to neighbouring farms.

Dung heaps, potential breeding sites of some Obsoletus Complex species, existed on all cattle and horse farms within a 50–100 m radius to the outdoor traps. Stables were cleaned on a daily basis, except for the sheep stable and cattle stable 1. The sheep stable was a deep-straw bedding stable, which was only cleaned after the end of the sampling period after 4 years of usage. Cattle stable 1 was a freestall and was cleaned once during the collection period in early February 2015.

Owing to only one sheep farm examined and the differing conditions between this farm and the other farms (no neighbouring farms close by, surrounded solely by fields, draughty location, no dung heaps and deep-straw bedding type), the results obtained from the sheep farm were considered “additional” and are therefore often discussed separately.

### Biting midge collection

For the collection of biting midges, two BG-Sentinel UV-light traps (Biogents, Regensburg, Germany) were operated on every farm. One trap was installed inside each stable, the second outside, at approximately 2 m height with its trapping hole. Both traps were located in close distance to a permanent stable opening. At the same time, the inside traps were placed next to the potential hosts, the outside traps right at the stable building and also in close distance to the host animals (<30 m). All traps were run once a week for 24 h, with both traps per farm being operated simultaneously. Sampling only took place when weather conditions were suitable for midge activity (no heavy rain or wind).

A Hobo Pro v2 data logger (Onset Computer Corporation, Bourne, MA, USA) was placed right beside each indoor insect trap (at the same height within approximately 30 cm) to record the inside temperatures every 4 h during the entire sampling period. Outside temperatures were obtained from two permanent weather stations in 2 m height (region 1: Airport Berlin-Brandenburg N 52.38, E 13.52; region 2: Müncheberg N 52.52, E 14.12).

Insects were caught and stored in ethanol (75%) until morphological identification to species or complex level under a stereomicroscope using the keys of Delécolle (1985) and Mathieu et al. (2012). Species represented by only a few individuals were further subjected to molecular analysis by COI barcoding using primers PanCuli-COX1-211F and PanCuli-COX1-727R, as described by Lehmann et al. (2012).

In the following analysis, the term “threshold temperature” marks the mean temperature of the 7 days prior to the first measured midge activity at a site or of a specific culicoid species. The mean weekly temperature instead refers to the average temperature of the named calendar week.

## Results

Throughout the sampling period, 160 trap catches were made. A total of 3667 ceratopogonids were collected of which 3465 (94.5%) belonged to the genus *Culicoides*. 3372 of the culicoid midges were females (97.3%), and 80.5% (*n* = 2790) were members of the Obsoletus Complex (herein considered consisting of the species *Culicoides obsoletus* (Meigen), 1818, *Culicoides scoticus* Downes and Kettle, 1952, *Culicoides chiopterus* (Meigen), 1830 and *Culicoides dewulfi* Goetghebuer, 1936). The remaining 675 specimens represented six additional culicoid species (Table 2).

**Table 2:** Total numbers and species composition (in percentage) of sampled *Culicoides* inside and outside of each stable

Species/species group	Host stable															
	Cattle 1		Cattle 2		Cattle 3		Horse 1		Horse 2		Horse 3		Sheep		Total	% of total
	Inside	Outside	Inside	Outside	Inside	Outside	Inside	Outside	Inside	Outside	Inside	Outside	Inside	Outside		
Obsoletus Complex	428	478	235	98	668	316	155	58	9	79	136	102	25	3	2790	80.52
*Culicoides deltus*	3	1			1	1									6	0.17
*Culicoides pictipennis*				1											1	0.03
*Culicoides poperinghensis*					2										2	0.06
*Culicoides pulicaris*	6	3		1	176	122									308	8.89
*Culicoides punctatus*	45	48	19	19	135	48	12	5		1	14	3	2	1	352	10.16
*Culicoides riethi*	1	4		1											6	0.17
Total	483	534	254	120	982	487	167	63	9	80	150	105	27	4	3465	100.00

More *Culicoides* specimens (59.8%) were collected inside than outside of the animal stables, equating a ratio of 1.6:1 (2072 indoors vs. 1393 outdoors). In total, the three cattle farms revealed almost five times as many sampled individuals (*n* = 2860) as the three horse farms (*n* = 574). Only 31 specimens were caught on the sheep farm (Table 2).

### Species composition

The collections also show a higher number of midge species on cattle farms than on horse farms. On cattle farms, seven culicoid species could be found: the Obsoletus Complex (for this analysis regarded as one species), *Culicoides pulicaris* (Linnaeus), 1758, *Culicoides punctatus* (Meigen), 1804, *Culicoides deltus* Edwards, 1939, *Culicoides riethi* Kieffer, 1914, *Culicoides pictipennis* (Staeger), 1839 and *Culicoides poperinghensis* Goetghebuer, 1953. In comparison, only two species were collected on horse farms and the sheep farm: *C. punctatus* and Obsoletus Complex (Table 2).

Except for *C. pictipennis*, all collected culicoid species were found inside animal stables. Six species were sampled outdoors: Obsoletus Complex, *C. deltus*, *C. pictipennis*, *C. pulicaris*, *C. punctatus* and *C. riethi* (Table 2).

### First seasonal midge activity

Midge activity could be observed as early as during the 12th calendar week inside the sheep stable and 1 week later inside all three cattle stables (Table 3).

**Table 3:** Time, species and temperature of the first seasonal midge activity at the various collection sites

Farm	Site	Week of first activity	Species sampled during first midge activity	Mean (lowest) temperature of 7 days prior to first activity	Mean (min/max) temperature on day of first activity
Horse 1	Inside	16	Obsoletus Complex	11.8 (7.4)	9.0 (6.1/10.8)
	Outside	17	Obsoletus Complex	10.1 (−1.1)	15.3 (7.6/23.0)
Horse 2	Inside	17	Obsoletus Complex	11.2 (4.8)	10.6 (7.6/14.2)
	Outside	17	Obsoletus Complex, *Culicoides punctatus*	9.4 (−1.1)	10.3 (1.4/18.7)
Horse 3	Inside	16	Obsoletus Complex, *C. punctatus*	12.3 (6.5)	9.3 (5.6/12.2)
	Outside	16	Obsoletus Complex	9.6 (−0.8)	8.3 (−1.1/15.2)
Cattle 1	Inside	13	Obsoletus Complex	8.4 (3.0)	12.9 (11.6/14.9)
	Outside	16	Obsoletus Complex	10.9 (−0.3)	10.4 (4.2/13.9)
Cattle 2	Inside	13	Obsoletus Complex	7.4 (2.8)	12.0 (11.1/13.6)
	Outside	16	Obsoletus Complex	10.9 (−0.3)	10.4 (4.2/13.9)
Cattle 3	Inside	13	Obsoletus Complex	9.9 (3.9)	14.9 (13.2/16.1)
	Outside	16	Obsoletus Complex, *Culicoides pulicaris*, *C. punctatus*	10.9 (−0.3)	10.4 (4.2/13.9)
Sheep	Inside	12	Obsoletus Complex	Not available	16.2 (14.1/20.4)
	Outside	19	Obsoletus Complex, *C. punctatus*	13.1 (−0.2)	12.4 (5.1/17.5)

Members of the Obsoletus Complex were the first midges caught at each collection site. At some sampling sites, where midge activity started during week 16 or later (inside all horse stables and all outdoors sites), species like *C. punctatus* and *C. pulicaris* were occasionally present among the first active culicoids in addition to the Obsoletus Complex (Table 3).

The minimum and maximum temperatures recorded during the entire time of sampling (including all study sites) were −8.2 °C (weather station Airport Berlin-Brandenburg, 7 February 2015) and 26.9 °C (horse stable 2 indoors, 1 May 2015).

The threshold temperatures at the various sites were between 7.4 and 13.1 °C (Table 3) with a total mean threshold temperature of 10.5 °C for all collection sites (except sheep stable indoors due to missing data). The lowest temperatures within 7 days prior to the first sampled midge activity per site was often sub-zero, once even as low as −1.1 °C (Table 3).

The average (minimum/maximum) temperatures of the day of the first measured midge activity per collection site varied from 8.3 to 16.2 °C (Table 3), resulting in a total mean daily temperature of 11.6 °C for all trap locations.

### Onset of seasonal midge activity regarding species

The first seasonally active species were midges of the Obsoletus Complex, followed by *C. pulicaris* and *C. punctatus* (Table 3) and, with some delay, by other culicoid species. This chronological order is also apparent in the species-specific threshold temperature pattern as illustrated in Table 4. Though no threshold temperature can be given for Obsoletus Complex (due to missing data), *C. punctatus* and *C. pulicaris* specimens started to show activity when the threshold temperatures rose up to 10.9 °C. Based on the early start of activity of Obsoletus Complex midges, their threshold temperature is therefore likely to be lower than 10.9 °C. Other culicoid species were collected when the threshold temperatures were much higher and reached 13.1, 15.1 and 16.9 °C (Table 4).

**Table 4:** Time and temperature of first midge activity per species

Species	Week of first activity	Species-specific threshold temperature^a^ (°C)	Minimum temperature of 7 days prior to first activity (°C)	Lowest min (mean/max) temperature during positive sampling days (°C)
Obsoletus Complex	12	No data	No data	−1.1 (8.3/15.2)
*Culicoides punctatus*	16	10.9	−0.3	1.4 (10.3/18.7)
*Culicoides pulicaris*	16	10.9	−0.3	2.6 (12.5/20.7)
*Culicoides deltus*	18	15.1	8.4	5.1 (12.4/17.5)
*Culicoides pictipennis*	19	13.1	−0.2	5.1 (12.4/17.5)
*Culicoides riethi*	19	13.1	−0.2	5.1 (12.4/17.5)
*Culicoides poperinghensis*	19	16.9	10.8	15.8 (18.7/22.0)

^a^If the first activity started simultaneously at different sites, only the lowest temperatures were considered

Except for *C. deltus* and *C. poperinghensis*, below zero temperatures were recorded within 7 days prior to the species’ first activity (Table 4).

When considering only sampling days with culicoid activity, the lowest minimum temperature was −1.1 °C (week 16, horse stable 3 outdoors). The associated daily mean temperature was 8.3 °C when midges of the Obsoletus Complex were collected (Table 4).

**Fig. 1:**
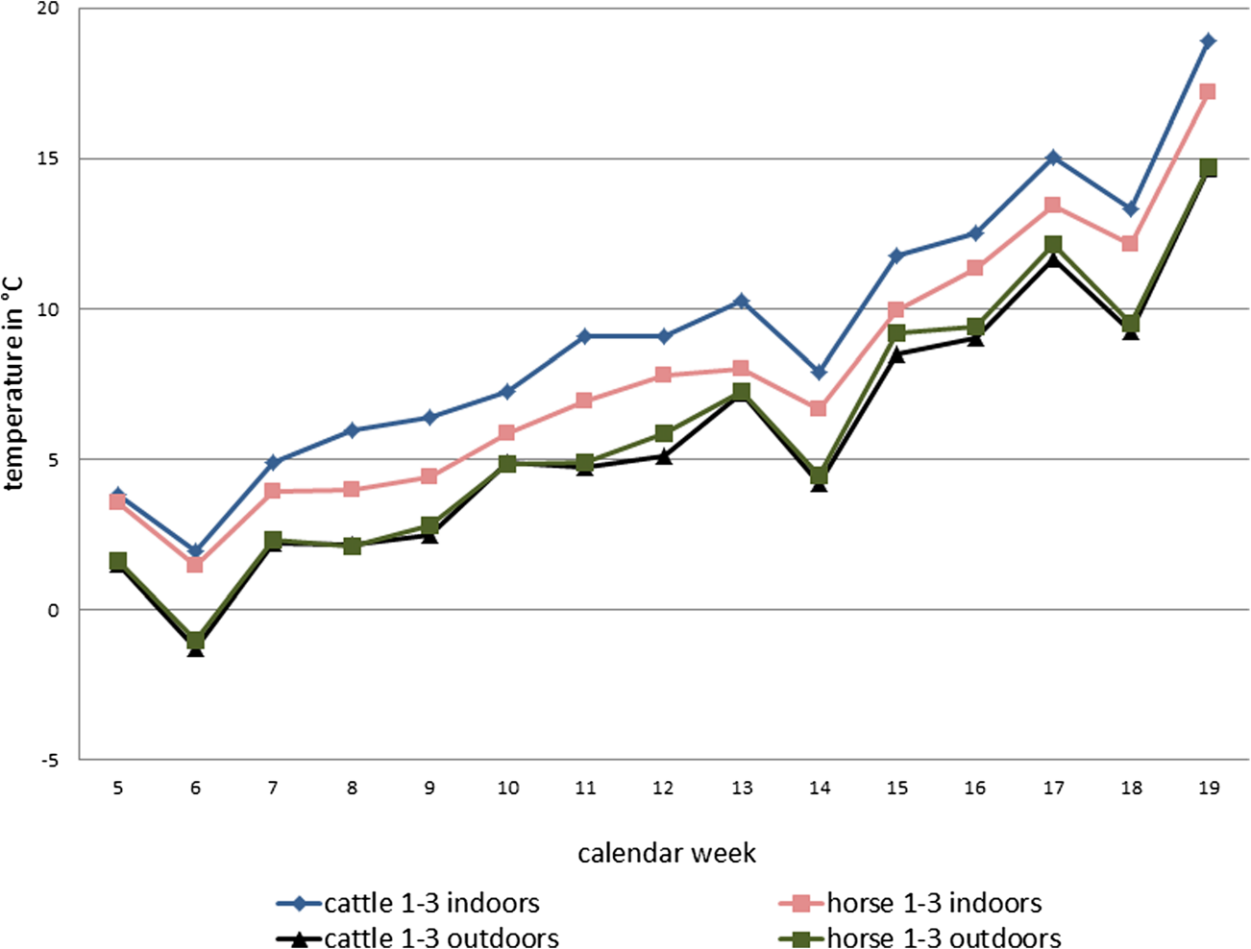
Mean weekly temperatures measured inside and outside of the cattle and horse stables in calendar weeks 5–19, 2015

As expected, the mean weekly temperatures recorded inside stables were higher than the corresponding temperatures outdoors. As illustrated in Fig. 1, they were highest inside the cattle stables (mean value for cattle stables 1–3), lower inside the horse stables (mean value for horse stables 1–3) and almost identically low outside of the cattle and horse farms (mean value for farms 1–3) (Fig. 1).

### Endophily/exophily

**Fig. 2:**
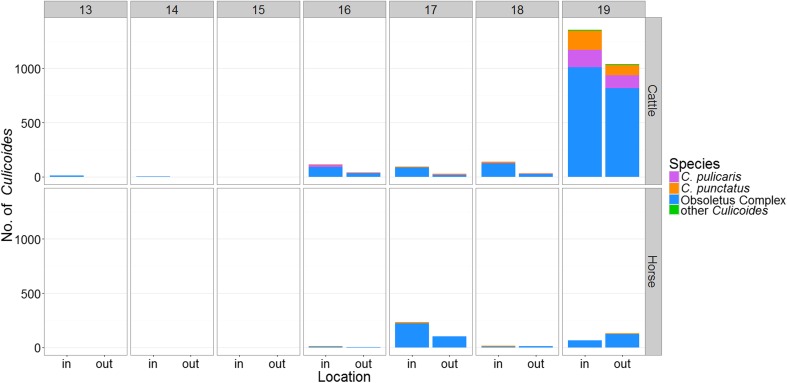
Average number of caught midges in calendar weeks 13–19 inside and outside of cattle farms 1–3 (*top*) and of horse farms 1–3 (*bottom*)

Activity of *Culicoides* started inside cattle stables in week 13, but the number of collected specimens remained on a low level until week 16, when midges began to show activity outside cattle stables as well. An explosive increase in midge numbers was measured in week 19, the last week of sampling. This increase was mostly caused by a much higher number of Obsoletus Complex midges, though in that week, *C. punctatus* and *C. pulicaris* were caught in relevant numbers, too. All three species were more abundant inside cattle stables throughout the entire sampling period which led to a higher total indoor number of *Culicoides* (Fig. 2).

On horse farms 1–3, the first midge activity was measured in week 16 indoors and outdoors with very low specimen numbers. A temporary increase of both indoor and outdoor numbers took place in week 17 with the highest recorded number of specimens of the entire sampling period. Until week 18, more *Culicoides* were collected indoors than outdoors. Week 19 showed decreased numbers. Besides the Obsoletus Complex, which was by far the dominant species on horse farms 1–3, only *C. punctatus* was sampled in low numbers. This species appeared mostly indoors until week 18. Only in week 19 *C. punctatus* was caught in higher numbers outside the horse stables than indoors (Fig. 2).

## Discussion

In the present study, we revealed a vector-free period inside and outside of stables which lasted until mid-march, 2015 (week 12). Some studies, usually conducted in countries with a milder climate (Romón et al. 2012), during milder winters (Losson et al. 2007; Mehlhorn et al. 2009a, b) or inside warm stables (Losson et al. 2007) were able to detect a constant culicoid activity throughout winter time, revealing a possible pathway of how *Culicoides*-borne arboviruses might survive the cold winter months in temperate regions. As ambient air temperatures during the time of sampling are considered to be the crucial factor to regulate midge activity (Lühken et al. 2015), a more detailed examination of the recorded temperatures is required when investigating the winter phenology of *Culicoides*.

### First seasonal midge activity

Losson et al. (2007) showed that adult midges can remain active inside stables during winter due to a milder indoor climate. Therefore, it seemed likely that after the midge-free period in the present study, culicoid activity could be found inside stables first. Our results partly confirm the hypotheses of an early indoor activity, with midges being sampled inside the sheep and all three cattle stables. But the simultaneous start of midge activity inside the horse stables and at all outdoor sites seems to question this explanation and implies that other factors might have delayed the start of culicoid activity inside the (warmer) horse stables. We assume, that most midges at horse farms develop outdoors and, once temperatures are high enough, emerge and become active outside of the stables. Some of the active culicoids might then also enter the horse stables, leading to an almost equal number of specimens collected indoors and outdoors in week 16 in the present study (Fig. 2).

A comparison of indoor and outdoor sampling sites revealed that the mean weekly threshold temperatures were slightly above 10 °C, which is in accordance to the temperatures determined under laboratory conditions (Lühken et al. 2015).

Should our theory be correct though, that midges did not develop inside horse stables in bigger numbers, the corresponding threshold temperature for inside horse stables should not be considered. Instead, the correlated outdoor temperature of 9.7 °C (outside horse stables 1–3) seems to be the true threshold temperature for midge activity on the horse farms in the present investigation. As the lowest threshold temperature of all sampling sites was 7.4 °C (inside cattle stable 2), this field-measured temperature seems to provide sufficient warmth to initiate midge activity.

Quite often, minus temperatures were recorded within 7 days before the first midge activity took place (Table 3). Even during the day of the first measured midge activity, temperatures were as low as −1.1 °C (Table 3) which proves that even below zero degrees do not prevent midge development or adult culicoid activity, as long as the freezing is only short-termed.

### Species composition

The culicoid species composition found in the present study (including all sampling sites) equates the species composition stated for most of Germany with the Obsoletus Complex being represented by 70% to >90%, the Pulicaris Complex by up to 20% of the present *Culicoides* and only relatively few specimens belonging to other culicoid species (Hörbrand and Geier 2009; Mehlhorn et al. 2009a, b). In this context, it was irrespective of the trapping sites being located indoors or outdoors. Therefore, the overall species composition seems not to be influenced by possible (temporary) endophilic/exophilic tendencies of some culicoid species in the present study.

Species composition and the number of collected midges differed strongly between livestock animals though, with cattle farms representing the most culicoid species and highest specimen numbers compared with all horse farms and the sheep farm (Table 2).

The extremely low number of sampled *Culicoides* on the sheep farm might be explained by the isolated and windy location of the farm. The differing species composition and midge numbers between cattle and horse farms might be caused by variant husbandry practises. Better clean outs of horse stables for example, might lead to a lack of microhabitats, which might be present only in cattle stables, e.g. dried cattle dung adherent to walls as proposed by Zimmer et al. (2010). Also, the better quality of the moist cattle dung as a potential breeding substrate as compared with the drier horse and sheep droppings might add to the higher number of Obsoletus Complex specimens being caught on cattle farms as described by Thompson et al. (2013). Undetected breeding sites in the surroundings of cattle farms cannot be ruled out as well. As all study farms and their neighbourhoods had been inspected carefully before the start of the study and considered as “comparable with each other”, we consider this explanation as relatively unlikely, though. Based on our own experience, differences in the species composition between the two sampled regions do not exist as well.

Blood host preferences could also have had an impact on the distribution of some culicoid species between farms. Even though Obsoletus Complex midges are known to be the generalists in the genus *Culicoides* (Lassen et al. 2012), it cannot be ruled out that other culicoid species which are represented only by a few individuals and which have not sufficiently been analysed in regards to their blood feeding preferences, might just not be present on the horse or sheep farms because these livestock animals do not belong to their preferred range of blood hosts.

### Onset of seasonal activity regarding species

In the present study, the species-specific threshold temperatures, triggering midge activity, differ widely (Table 4). While *C. punctatus* and *C. pulicaris* started to show activity after their threshold temperatures reached nearly 11 °C, the threshold temperatures for *C. deltus, C. pictipennis, C. riethi* and *C. poperinghensis* seem to be higher. Therefore, it may be possible, that these species may have appeared on horse farms, too, once temperatures have reached the species’ threshold temperatures after the end of the sampling period. As only a low number of farms were sampled and only few midge specimens per species were collected, the species-specific threshold temperatures should be interpreted with care and provide only a first baseline dataset for future studies.Except for *C. poperinghensis* and *C. deltus*, all collected species were able to resist short-termed minus degrees, when minimum temperatures (within 7 days prior to their first activity) went down to below zero (Table 4).

### Endophily/exophily

In our investigation, the total number of sampled *Culicoides* was higher indoors than outdoors with a ratio of 1.6:1, which may be attributed to the higher indoor temperatures. Previous studies proved a strong exophily for culicoid midges (Baldet et al. 2008; Baylis et al. 2010; Meiswinkel et al. 2008) and also described varying underlying conditions in their investigations, e.g. livestock movements between indoor and outdoor resting places (Meiswinkel et al. 2008) or higher outdoor temperatures caused by different sampling seasons (Baylis et al. 2010; Meiswinkel et al. 2008). Baldet et al. (2008) assumed that *Culicoides* are neither purely endophilic nor exophilic but may behave according to outdoor temperatures. The results of the present investigation show a noticeable preference for indoor locations at least during the coldest time of the year (Fig. 2). Nevertheless, the fact that more Obsoletus Complex midges were caught outside of horse stables in week 19 suggest, that their habitat preferences are not rigidly predetermined, but a reaction to environmental factors, conceivably temperature, as proposed by Baldet et al. (2008). According to our findings, the adaptation to endo−/exophilic behaviour does not only account for Obsoletus Complex midges, but also for *C. punctatus*. Though *C. punctatus* was only sampled in small numbers on horse farms, it was caught in higher numbers inside than outside horse stables except for week 19 when more specimens were collected outdoors (Fig. 2). The total numbers of collected *C. punctatus*, but also of *C. pulicaris*, reveal a marginal endophilic tendency of these species at least during the cold winter months. This is contrary to previous results published by Baldet et al. (2008) and Meiswinkel et al. (2008), who illustrated a strong exophilic behaviour for both species.

Other species like *C. riethi* and *C. deltus* were only represented by six individuals each. Due to the low number of specimens, it is impossible to evaluate, if the observed endophilic tendency for *C. deltus* and the exophilic tendency for *C. riethi* represent an actual pattern of behaviour.

Regarding endophily and exophily of the various biting midge species, it needs to be taken into account that only three horse farms, three cattle farms and one sheep farm were investigated. Thus, the data should be considered preliminary. They may illustrate the complexity of the midges’ behaviour and phenology and demonstrate the necessity for more research.

## Conclusion

We found a vector-free period in our investigation lasting until mid-March, 2015. We showed that the first seasonal biting midge activity was initiated by a threshold temperature of at least 7.4 °C and that even below zero temperatures did not prevent midge development or adult midge activity, as long as the freezing was only short-termed.

The type of livestock animals seemed to influence the starting time of midge activity, the species composition and the numbers of midges collected. This may be attributed to differences in ambient temperature and, most likely, also to husbandry practises. Midges of the Obsoletus Complex were the first to become active.

Species composition hardly differs between indoor and outdoor trapping sites during early spring as most species were present both indoors and outdoors. This suggests that they are not strictly endophilic or exophilic, but that behaviour is influenced by environmental factors.
